# Supplementary figures and images for: Host nutritional status affects alphavirus virulence, transmission, and evolution
Source: PLoS Pathog. 2019 Nov 11;15(11):e1008089. doi: 10.1371/journal.ppat.1008089 (PMC6872174; doi:10.1371/journal.ppat.1008089)

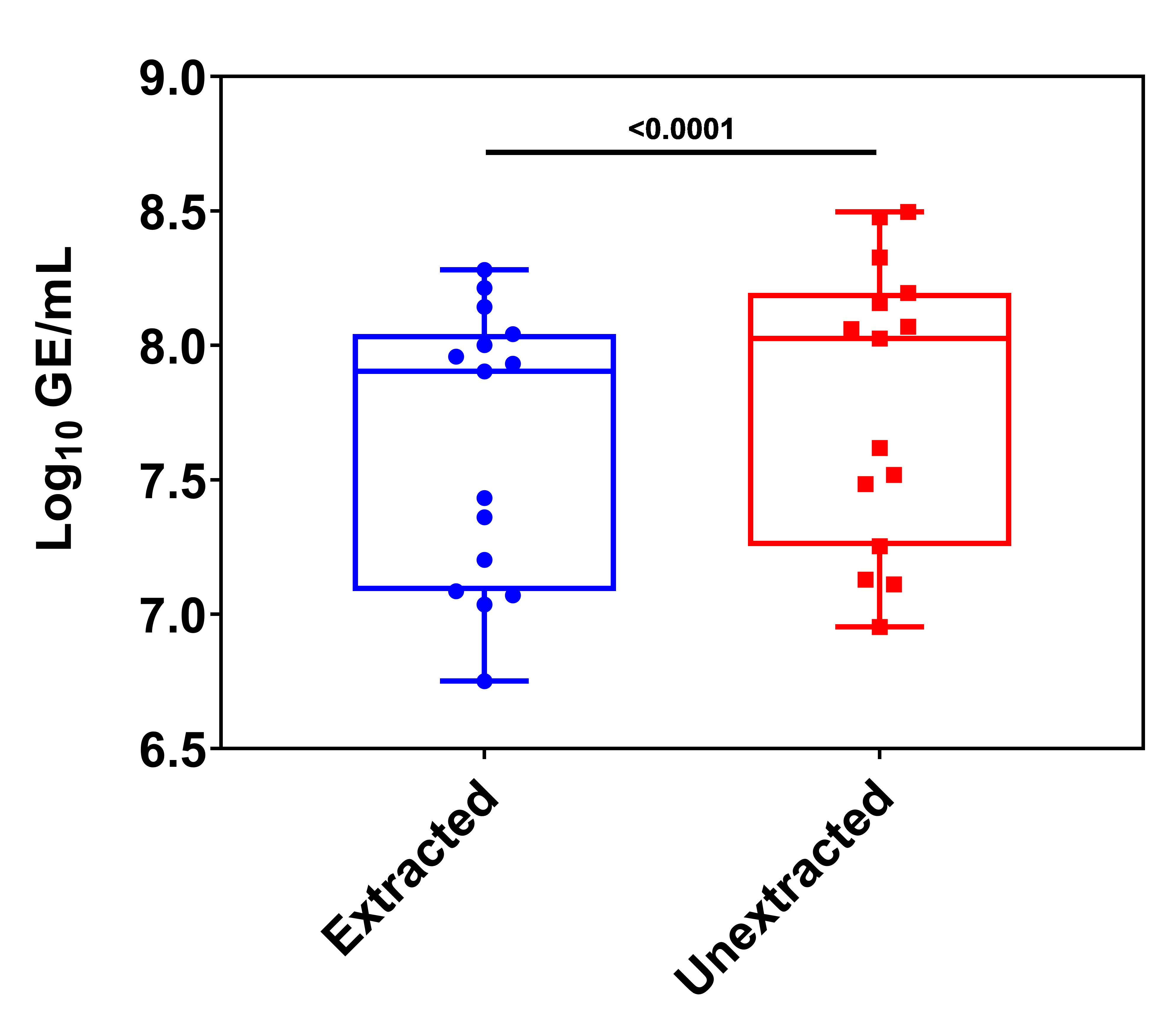

Supplement: S1 Fig — Serum samples from mice infected with Mayaro virus (MAYV) were used to assess the necessity for an RNA extraction step before one-step quantitative reverse-transcriptase PCR (qRT-PCR). qRT-PCR was performed using the NEB Luna Universal Probe kit with MAYV specific primers. For RNA extractions, the Zymo Viral DNA/RNA extraction kit was used. For testing without extraction, the samples were diluted 1:5 in PBS and then used directly for qRT-PCR. Viral RNA prepared from an infectious clone was serially diluted in ten-fold dilutions and used to generate a standard curve. The sample values were then fit to this standard curve and the volumes normalized to calculate the number of genome copies per mL of serum (genomes/mL). The whiskers represent the minimum and maximum points, the box limits represent the 25th and 75th percentile, and the horizontal line represents the median. Statistical comparison was performed using a two-tailed paired t-test. (TIF) [file ppat.1008089.s001.tif]

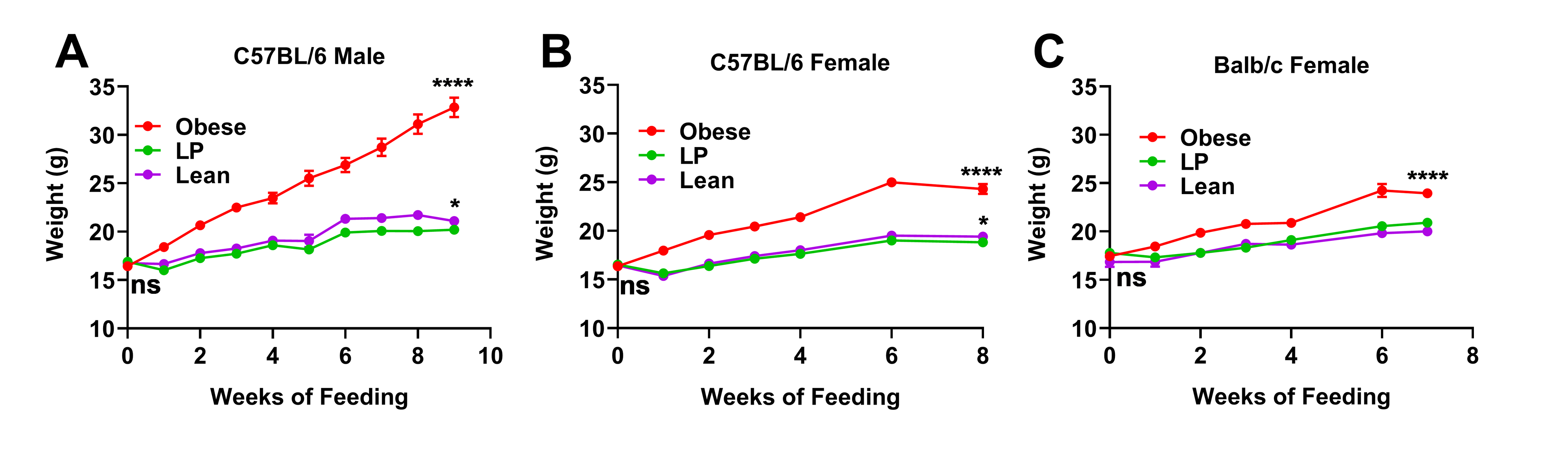

Supplement: S2 Fig — Groups of C57BL/6N (A-B, 7–14 mice per group) or Balb/c (C, 5 mice per group) were fed a 45% fat (Obese), 5% protein (LP), or a control (Lean) diet for 8–10 weeks. Weights were measured weekly or bi-weekly following the initiation of feeding. Weights are plotted in grams. Statistical comparisons were made using a two-way ANOVA with Dunnett’s correction compared to the control group. The level of significance is represented as follows–ns (not significant) p>0.05, * P<0.05, **** p<0.0001. The **** represents the comparison between the lean and obese groups. The * represents the comparison between the lean and the low protein groups. The error bars represent standard error. Each graph represents data obtained from at least two independent experiments except for C, which was performed once. The results from one representative experiment are presented. (TIF) [file ppat.1008089.s002.tif]

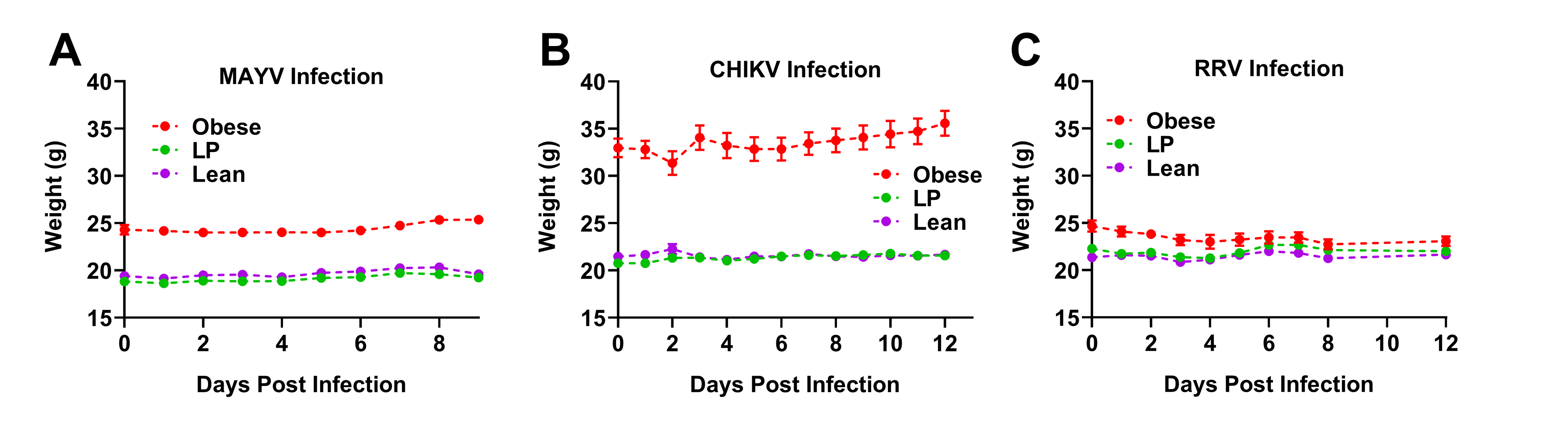

Supplement: S3 Fig — Groups of C57BL/6N (A-B, 7–14 mice per group) or Balb/c (C, 5 mice per group) were fed a 45% fat (Obese), 5% protein (LP), or a control (Lean) diet for 8–10 weeks. Mice were then infected with Mayaro virus (MAYV, A), chikungunya virus (CHIKV, B), or Ross River virus (RRV, C). Weights were measured daily following infection. Weights are plotted in grams. The error bars represent standard error. Each graph represents data obtained from at least two independent experiments except for C, which was performed once. The results from one representative experiment are presented. (TIF) [file ppat.1008089.s003.tif]

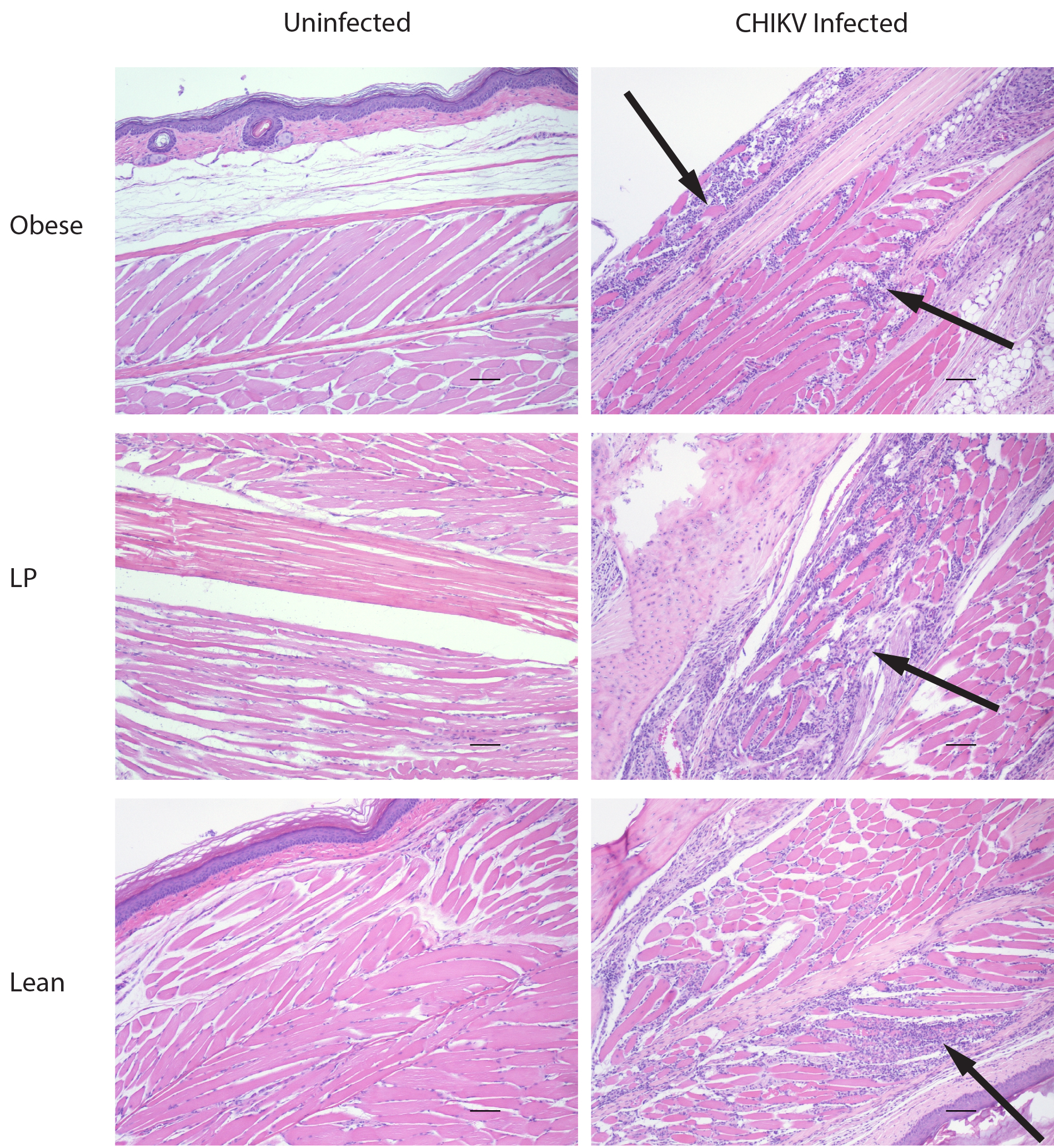

Supplement: S4 Fig — Groups of C57BL/6N mice were fed either 45% fat (Obese), 5% protein (LP) or control (Lean) diet for 8–10 weeks. Mice were then infected with chikungunya virus (CHIKV). Nine days post-infection, the left hind footpad was collected and placed into formalin for fixation. The fixed tissues were then sectioned and stained with hematoxylin and eosin (H&E) and then scored in a blinded manner by two independent anatomic pathologists following the scoring guide presented by Hawman et al. 2013. Scale bar, 100 μM. Images are representative of 2 mice per group. The arrows represent areas of myositis. (TIF) [file ppat.1008089.s004.tif]

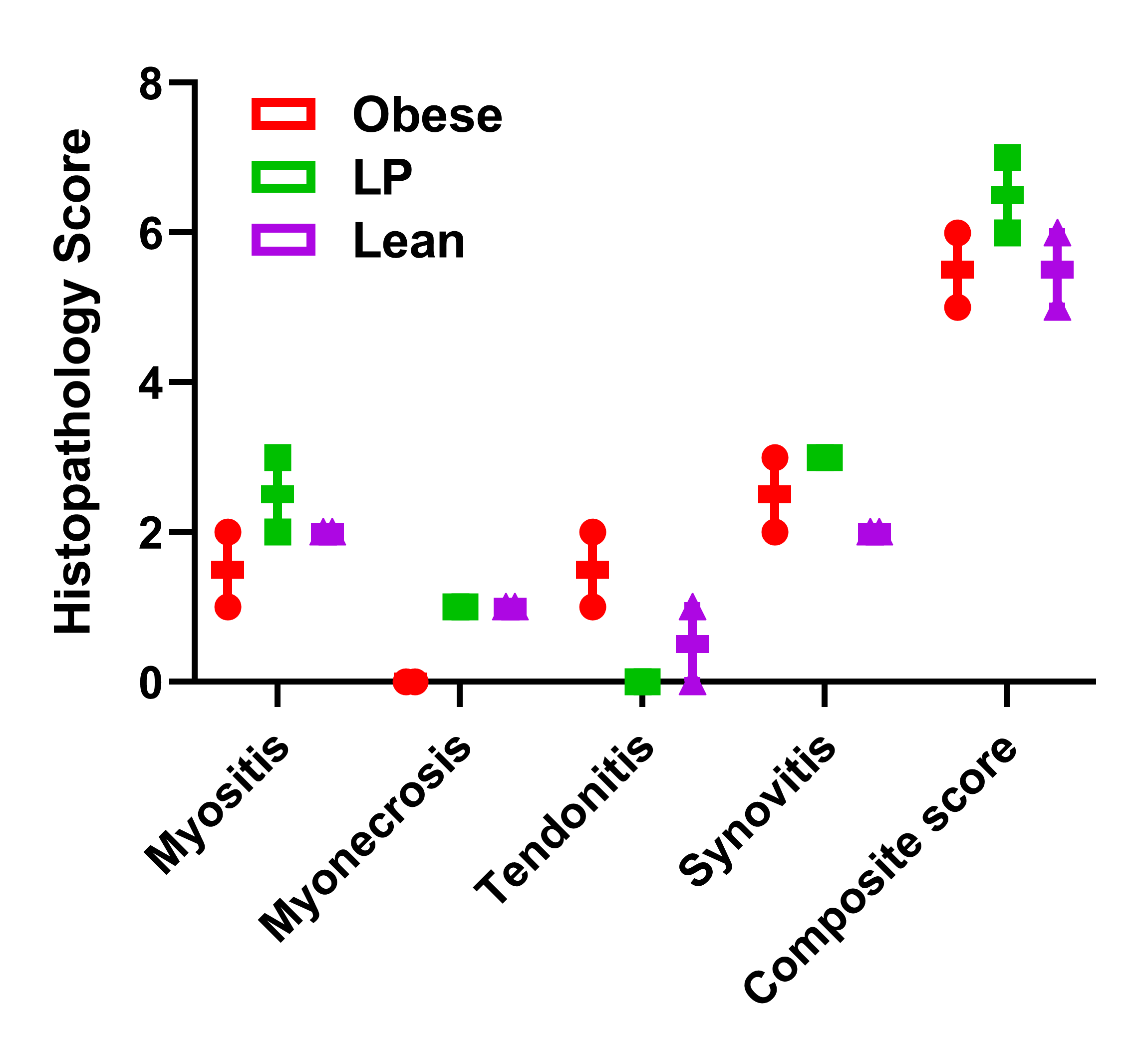

Supplement: S5 Fig — Groups of C57BL/6N mice were fed either 45% fat (Obese), 5% protein (LP) or control (Lean) diet for 8–10 weeks. Mice were then infected with chikungunya virus (CHIKV). Nine days post-infection, the left hind footpad was collected and placed into formalin for fixation. The fixed tissues were then sectioned and stained with hematoxylin and eosin (H&E) and then scored in a blinded manner by two independent anatomic pathologists following the scoring guide presented by Hawman et al. 2013. The middle bar represents the median. (TIF) [file ppat.1008089.s005.tif]

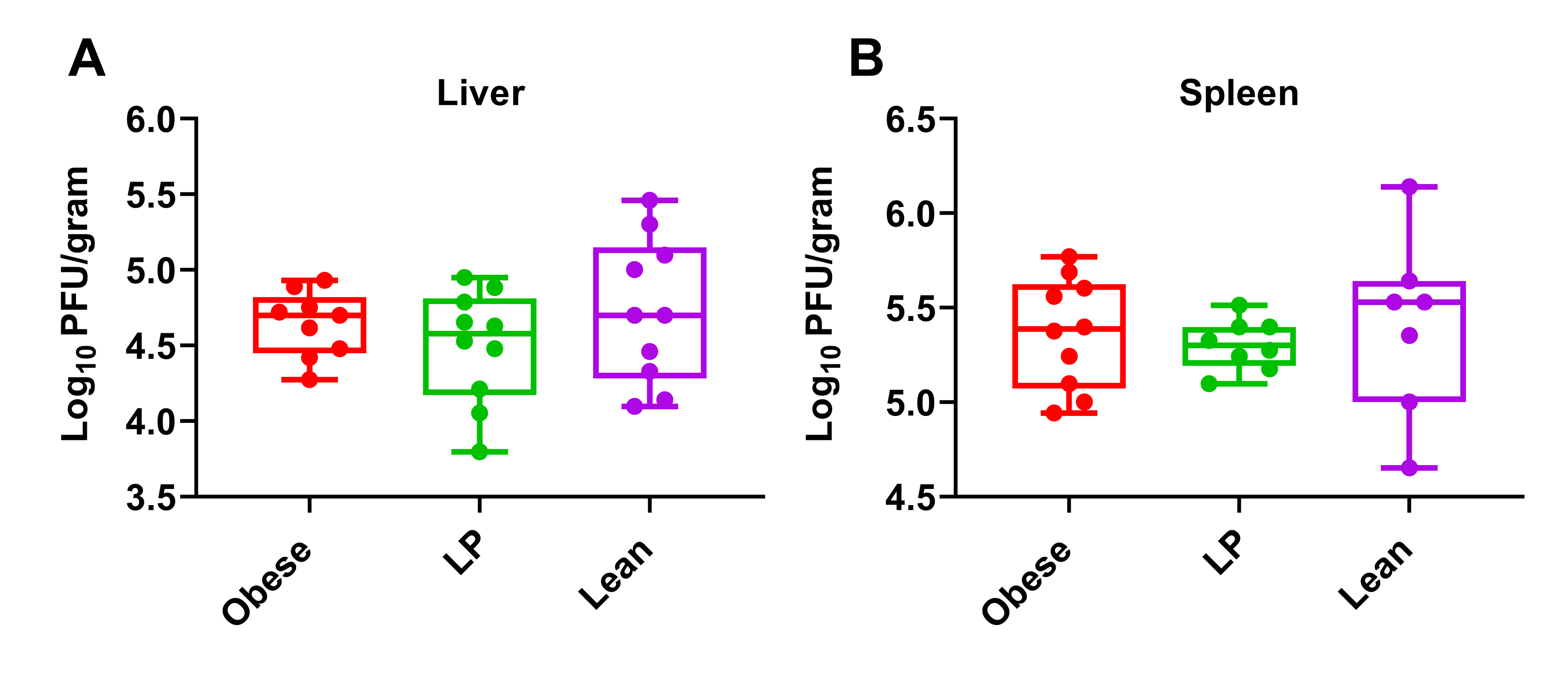

Supplement: S6 Fig — Groups of C57BL/6N mice (9–10 mice per group) were fed either a 45% fat (Obese), 5% protein (LP), or control (Lean) diet for 8–10 weeks. Mice were then infected with Mayaro virus (MAYV) and 2-days post-infection the liver (A) and spleen (B) were collected. Viral titer was measured in the tissue homogenates by plaque assay. The whiskers represent the minimum and maximum points, the box limits represent the 25th and 75th percentile, and the horizontal line represents the median. Statistical analyses were performed by one-way ANOVA with Dunnett’s correction compared to the control group. No statistically significant differences were found. (TIF) [file ppat.1008089.s006.tif]

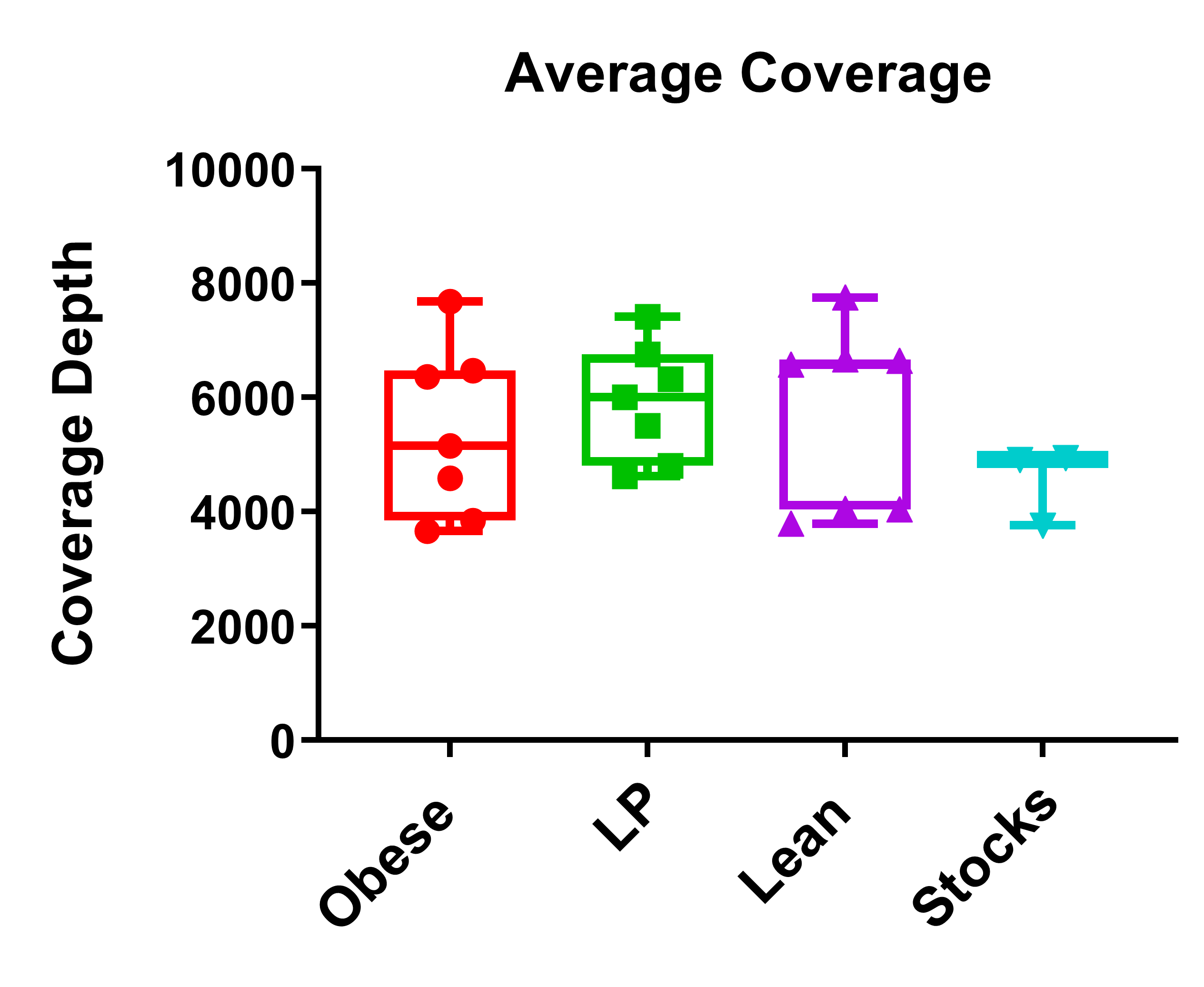

Supplement: S7 Fig — Groups of C57BL/6N mice (7 mice per group) were fed either a 45% fat (Obese), 5% protein (LP), or control diet (Lean) for 8–10 weeks. Mice were then infected with Mayaro virus (MAYV) and blood was collected two days later. RNA was isolated from the serum and next-generation sequencing libraries were prepared and then sequenced on an Illumina NextSeq 500. The FASTQ files were trimmed for adapter and low-quality sequences using BBDuk. The trimmed reads were then analyzed using the ViVAN pipeline with a coverage cutoff of >100x and a p-value filter of 0.05. From the output of ViVAN, we compared the sequencing coverage depth. The whiskers represent the minimum and maximum points, the box limits represent the 25th and 75th percentile, and the horizontal line represents the median. Statistical analyses were performed by one-way ANOVA with Dunnett’s correction and compared to the control group. (TIF) [file ppat.1008089.s007.tif]

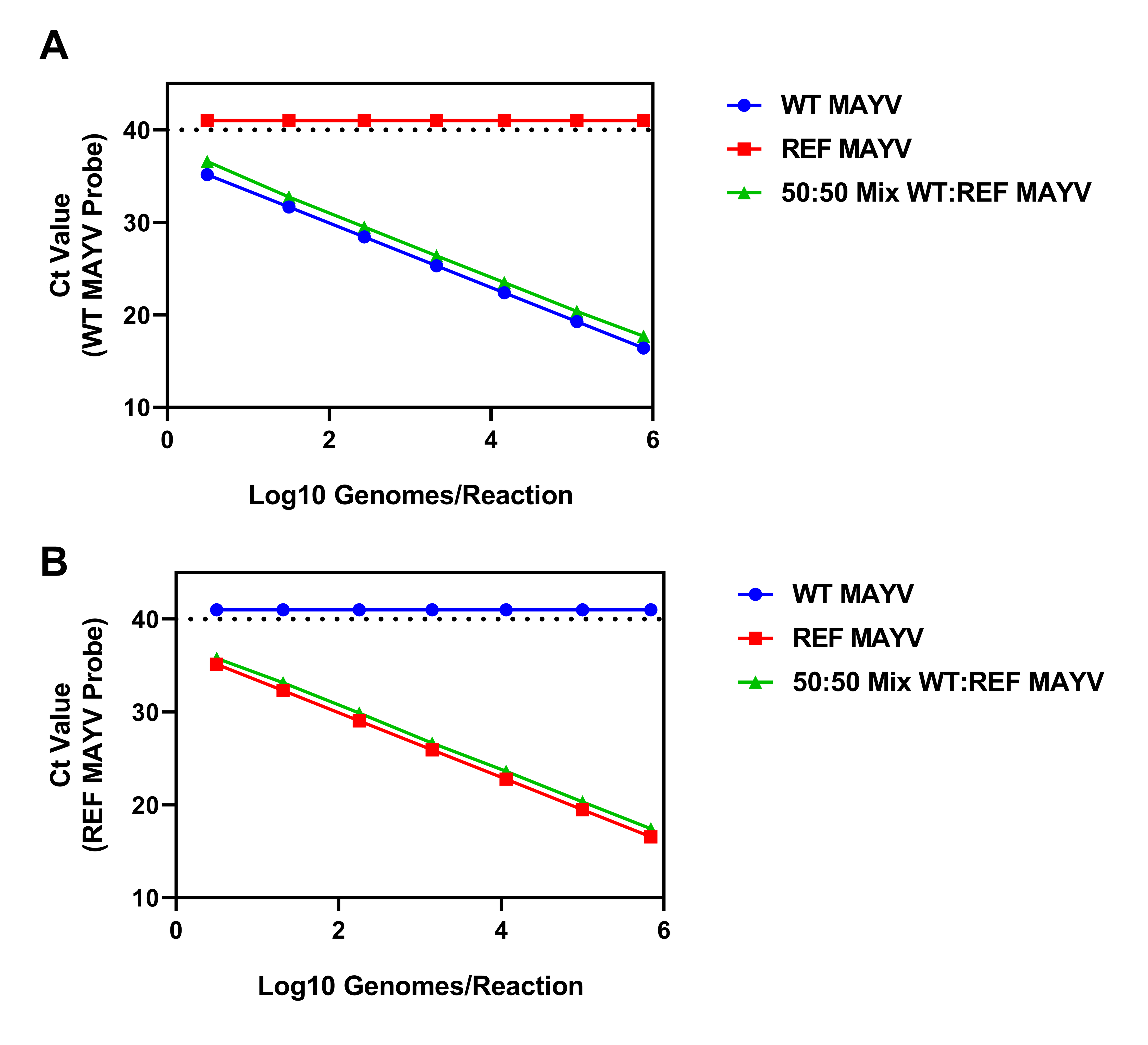

Supplement: S8 Fig — 10-fold dilutions of RNA derived from either wild-type (WT) or the marked reference (REF) Mayaro virus (MAYV) infectious clone plasmids were made. RNA was then mixed at a 1:1 ratio between WT MAYV and REF MAYV to simulate a mixed infection. Probe-based qRT-PCR was then performed with either a probe specific for WT MAYV (A) or REF MAYV (B). Forty cycles of PCR were performed. The dotted line represents the limit of detection. Any point that did not produce a Ct value was given an arbitrary Ct value of 41. (TIF) [file ppat.1008089.s008.tif]

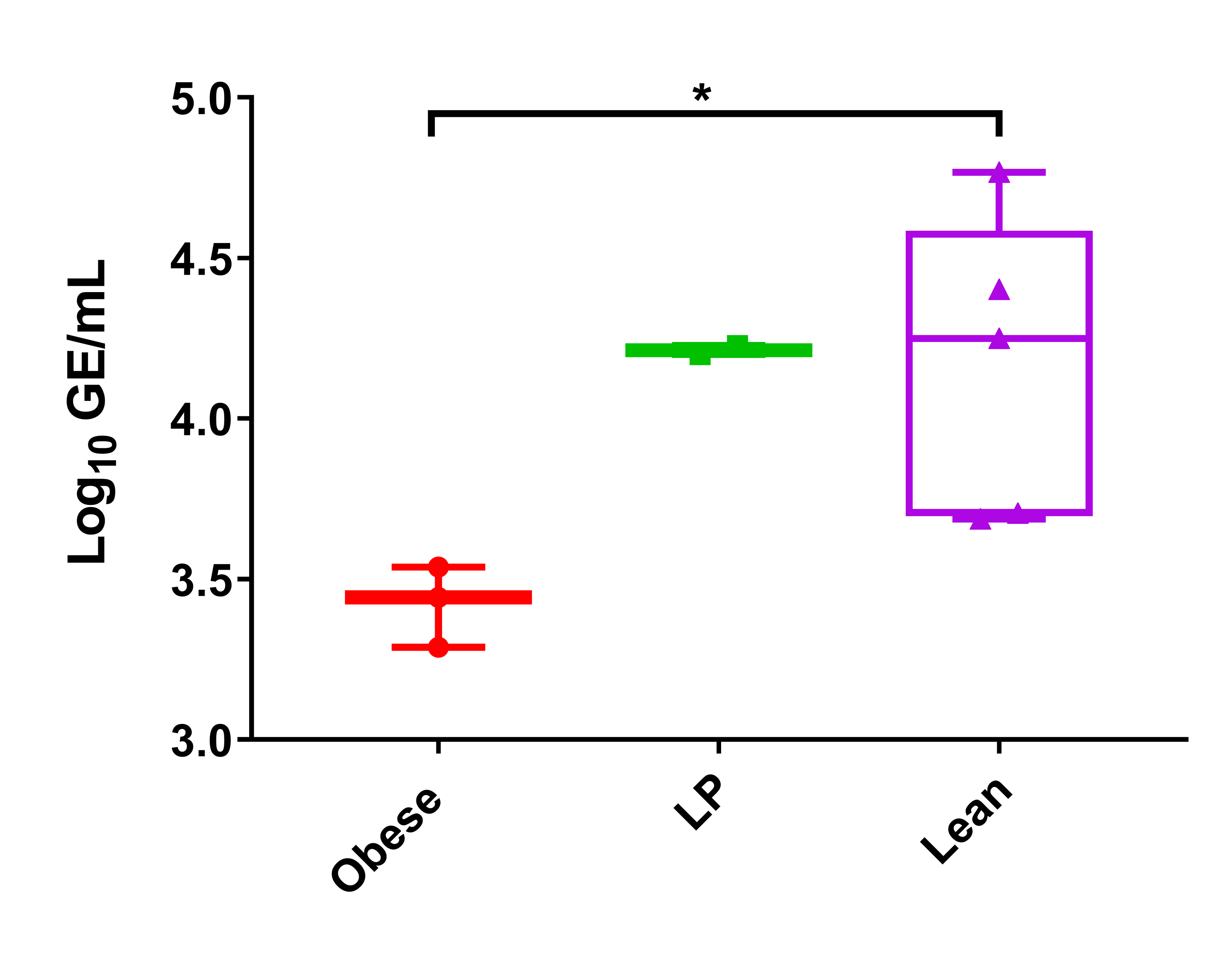

Supplement: S9 Fig — Groups of C57BL/6N mice (7 mice per group) were fed either a 45% fat (Obese), 5% protein (LP), or control (Lean) diet for 8–10 weeks. Mice were then infected with Mayaro virus (MAYV) and footpads were collected 65 days post-infection. Viral RNA was quantified using qRT-PCR. The whiskers represent the minimum and maximum points, the box limits represent the 25th and 75th percentile, and the horizontal line represents the median. Statistical analyses were performed by one-way ANOVA with Dunnett’s correction compared to the control group. The level of significance is represented as follows—* p<0.05. (TIF) [file ppat.1008089.s009.tif]
