# Supplementary material for: Host nutritional status affects alphavirus virulence, transmission, and evolution
Source: PLoS Pathog. 2019 Nov 11;15(11):e1008089. doi: 10.1371/journal.ppat.1008089 (PMC6872174; doi:10.1371/journal.ppat.1008089)
Supplement: Attachment — Submitted filename: Response to Reviewers - PPATHOGENS-D-19-01146.pdf [file ppat.1008089.s012.pdf]

Dear Drs. Kuhn, Heise, Haldar, and McFadden,

We are deeply grateful for your consideration of our manuscript "Host Nutritional Status Affects Alphavirus Virulence, Transmission, and Evolution" (Ref: PPATHOGENS-D-19-01146). We are indebted to the reviewers for their useful comments. We have taken their suggestions into account and made several changes to the manuscript which we believe have resulted in an improved submission. We have made changes to the discussion based on the comments from the reviewers which serve to improve readability. With these edits, we feel strongly that the manuscript will be appropriate for publication in *PLOS Pathogens*.

Below are the comments from the reviewers and our responses in bold.

Best Regards,
James Weger-Lucarelli

Part II – Major Issues: Key Experiments Required for Acceptance

Reviewer #1:

1. Supplementary Table 1 was not included with the manuscript. This table described the nutrient content of the diets, so this is critical missing piece. I am assuming this was just an oversight by the authors.
Yes, we are sorry about this oversight. We have included Supplementary Table 1 in our resubmission for your review.

2. In the introduction, the authors state that they fed mice two high fat diets-45% and 61%, but the data presented only includes 45% high fat diets. Did they use the 61% diet? And if so, did the results mirror the 45% diet? This would be important information to know.
We did use two high-fat diets in our first studies. We saw very similar results between the two diets and, therefore, proceeded with further studies with only the 45% high fat-diet. We chose the 45% diet since this seems like a more realistic percentage of fat as opposed to 61%. We have removed mention of the other diet from the manuscript.

3. Authors should present actual weights (figures 1 and 2) of mice rather than just percent loss or percent gain of weight. It can be added as supplementary data, but it needs to be there. The readers have no idea if the starting weights were identical, and how much weight in grams were lost or gained. How many grams were the obese mice?
Thank you for this suggestion. We have added the pre-infection weight, in grams, in supplementary figure 2. We have included a new supplementary figure 3 presenting the weight in grams following infection as well as suggested. This point has also highlighted an error in the previous manuscript. We stated that female mice were used for all studies. However, we used male mice in our studies with CHIKV and females in all of the rest of the studies. This has been updated in the materials and methods. As expected, male mice weighed more than females (~35g for males as compared to ~25g for females). There are stark differences in starting weight between the groups fed different diets before infection, es expected. Therefore, it is not possible to perform statistics on these values. The weight of the mice was dependent on the strain and the sex of the mice.

4. The authors mention diabetes in the discussion, but no metabolic markers were provided. What were the glucose levels of the mice? Were they, in fact, diabetic?

The mention of diabetes in the discussion has been removed. We did not measure glucose levels and therefore, cannot speculate on their status in terms of blood sugar.

5. The authors measure leptin, but it's unclear what the point is. It's well known that leptin levels increase with adiposity. They did not see a leptin difference between low protein and control diet-what is the significance of this finding? Does it matter? This data could be removed, unless the authors provide more context. It seems to be just "thrown in" as a piece of data.
We are sorry we did not provide sufficient context for these data. Leptin, as you mentioned, is strongly correlated with adiposity. It was included to highlight that the obese mice were genuinely obese. Other reports have suggested a reduction in leptin in mice fed a very low protein diet. However, our diet was not sufficiently low in protein to result in a significant reduction in leptin levels. Presenting leptin levels provides additional support that the obese mice are in fact, obese. These data are commonly presented in studies in obese mice and therefore feel that it may be informative for some individuals.

6. The legend for the stats on figure 1A is not clear. You have to read the text to understand the purpose of the one star that is shown on the data. The stats information should be clearly stated in the figure legend.
We agree with the reviewer that this was confusing. We have removed the star in Figure 1A and now have statistics presented in Supp. Fig 2, the new figure showing the weight change in grams over time before infection that was suggested by the reviewer.

7. With the CHIKV challenge, why were weight actually increasing after infection for the non-obese groups?
The CHIKV strain we used was not as pathogenic in this mouse model as MAYV or RRV, so these mice did not lose weight following infection. This highlights the effect of obesity on pathogenicity since the obese mice were the only group to present with weight loss.

8. For the pathology slides in Supp Fig 2, it would be helpful for the readers to use arrows to point out the inflammation, and to provide what histopathological score the photos represent.
Thank you for these useful suggestions. In the new Supp Fig 4 we have included arrows at sites of myositis. As suggested, we have also added a new Supp Fig 5 presenting histopathology scores.

9. The authors wanted to see if their results would be similar using a strain of mouse different from the B6 mice. This is a logical step, however, they also had to treat the mice with antibody to block type-I IFN-again, a major departure from the B6 mice. So this is not a good comparison between strains.
This is a good point. We are not truly comparing mouse strains but rather the effect of similar viruses in the context of nutrition in different mouse strains. We feel that the Balb/c model represents a suitable model in this case. Given that the results were consistent between the strains, this provides further support for our hypothesis that obesity results in more severe disease. We have removed any instance of potentially comparing between mouse strains.

10. In the discussion section, they claim that the model of RRV led to 60% of the undernourished mice and 40% of the obese died from the infection, yet all the lean mice lived. However, in the results section, they state that 2 mice from the obese group and 3 mice from the LP group died-out of a total of 7 mice. This is not 60% mortality. And, they also state that the

results were not statistically significant, so this does not rise to a level that needs to be discussed.
Thank you for catching this inconsistency. The percentages are correct, but we reported the incorrect group sizes in the legend for figure 2. We have updated the figure legend to reflect that the group size was 5 mice. As suggested by the reviewer, we have removed mention of the mortality from the discussion since it was not statistically significant.

Reviewer #2: 1) Type of fat included in the diet can be very influential on the outcomes of experimental infection. How was this particular diet chosen as a proxy for biological relevance? Also, it may be good include a genetically obese model in the experimentation to show that obesity itself and not diet, can result in similar changes as observed in these studies. This would also be a very interesting question to help cover the influence of leptin on outcomes of these experiments as the authors have chosen to use this measure as a proxy of nutritional status.
The diet selected was based on the standard in the field to induce obesity, a high-saturated fat diet. We have now included a new Supp Table 1, which provides a breakdown of the nutritional composition of each diet. The supplier (SAFE-diets) is a leader in supplying diets for rodents in France. For initial studies, we chose both a 45% and a 61% fat diet to induce obesity. The 45% fat diet used here is very similar in nutrient composition to a widely used diet sold by Envigo (TD.06415) to induce obesity in rodents. The validity of the diet was confirmed through the confirmation of the obesity of the mice used in these studies.

The suggestion to use genetically obese mice is a valid point and one that we considered in our experimental design. The ob/ob mice that it seems the reviewer is referring to is a mouse that lacks leptin. As you know, the vast majority of humans are not leptin deficient. Since leptin is known to function as an adipokine in addition to providing a signal for satiety, we considered this an unnatural model since most humans are obese due to diet. Therefore, we decided that diet induced obesity represented the most accurate depiction of obesity that occurs in the human population. While outside of the scope of this manuscript, we are indeed very curious about the role of leptin on alphavirus infection, which could be answered using the ob/ob mouse model. This is something we plan to pursue in future studies.

2) Since adipose tissue is a highly vascularized organ, it is possible that the virus is being trapped in the extra adipose and that is why the authors do not observe differences (or even lowered) viremia? Was any attempt made to measure viral particles in other tissues (especially adipose) aside from the footpad and serum? Similar studies would be interesting for the persistence of viral RNA. Please consider doing this experimentation.
This is an intriguing point. Yes, we did attempt to measure viral titers in both the spleen and liver. No differences were observed. As suggested, we have included this data in a new Supp. Fig 6.

Reviewer #3: None

Part III – Minor Issues: Editorial and Data Presentation Modifications
Please use this section for editorial suggestions as well as relatively minor modifications of existing data that would enhance clarity.

Reviewer #1: Some paragraphs in the discussion need to be broken up for better readability.

Thank you for this suggestion. We have broken up several paragraphs in an attempt to improve readability.

Reviewer #2:

1) In the introduction, the authors refer to four different diets (Line 90), but only discuss three diets in the feeding methodology described on lines 140-141. Please consider revising for consistency. The remainder of the paper only refers to the 45% diet. If data is available for the 61% diet, please include in the paper.
We did use two high-fat diets in preliminary studies. We saw very similar results between the two diets and, therefore, proceeded with repeat studies with only the 45% fat diet. We chose the 45% diet since this seems like a more realistic percentage of fat as opposed to 61%. We have removed mention of the other diet from the manuscript. We chose to not include data for the 61% fat diet since it was only included for studies with CHIKV and not the other viruses.

2) Please give references in the methodology text for successful employment of these diets in previous studies.
A reference for this diet has been included in the methods section as suggested. See line 144, reference 36.

3) Was any kind of clinical score (semi-quantitiative) taken during the mouse infections to monitor health of the animals? Hunching, coat quality, etc all could be monitored to increase knowledge of morbidity in these animals.
This is an excellent question. We were optimistic that we could generate this kind of data upon starting these studies. However, besides the mice treated with anti-IFNAR antibodies, none of the mice presented with hunching, ruffled hair, or any other differences that could be measured quantitatively. The immunocompetent nature of these animals in conjunction with the viral strains we used did not result in overt pathology other than mild weight loss and footpad swelling.

4) Lines 256: “weight gain” should just be “weight”?
Thank you for catching this. This has been corrected.

5) Please show all data from C57 versus Balb/c experimentation, including leptin measurements. This data is especially important as these animals are generally less susceptible to dietary treatment that the more widely used C57Bl/6 mice.
Thank you for pointing this out. While we did not measure leptin levels in Balb/c mice, we have now included the weights during feeding in Supp. Fig 2C. As you can see, weight gain was similar in both C57BL/6 and Balb/c, and both were significantly higher than the lean controls. There was no difference in weight between any group at the start of feeding.

6) The data from the serological viral titer determination and the luminescence studies seems to be contradictory. Was viremia/RNAemia measured at day 6 between obese and healthy models?
We measured only to 4 days post-infection in the serum of mice infected with CHIKV and 3 days post-infection for the other viruses. We did not include these data since the values stayed similar to 3 days post-infection, and we only had days 1-3 for all other studies. We do not feel these are necessarily contradictory results. It is possible that local replication at the site of infection could be increased but systemic replication is

decreased. This could be a result of reduced circulation in obese mice or some other factor. We do agree that this is an interesting point that were are currently following up on since this may be due to the immune response locally vs. systemically.

7) Is 2 days generally accepted as the period of time with the most changes in the viral quasispecies? This would be especially important to ask if a virus from a LP or obese mouse cause differential morbidity or mortality in a lean host aside from changes in fitness?
This is a good question. This time post-infection was selected since it was the peak of viral replication. On day 3, the viral titer dropped to such that transmission would be expected to be reduced considerably. Therefore, we chose 2 days post-infection as a marker of when the virus was most likely to be transmitted to a mosquito. It is possible that virus diversity would have shifted later in infection but this might not be as relevant since the virus would not be transmitted as efficiently. We are planning follow up studies to address the suggestion that the virus from either obese or LP mice can have altered pathogenicity in the lean host.

8) How do the genetic changes in the viruses correlate to pathogenicity of the viral species themselves aside from fitness?
We cannot speculate on this point specifically since we did not test this. In future studies, we would like to perform several passages in order to identify specific mutations that might arise in specific host environments (e.g. lean vs. obese). We attempted to find mutations that were specifically selected for in each environment but only found weak positive results. This is because after only one passage in a mouse, insufficient diversification occurs to find the signal from the noise. With additional passages, we expect that the signal will become much stronger.

9) While the LP diet is interesting (as it represents one of the main types of malnutrition, kwashiorkor), other types of malnutrition exist in areas endemic for these virsues including complete marasmus, caloric restriction, and micronutrient deficiencies. Can the authors further comment on how different types of undernutrition could further change the results of this study?
We have added additional discussion to address this point. The reviewer brings up a very valid point; malnutrition consists of many variations, each of which could have a different effect on pathogen-host interaction. See lines 394-395.

Reviewer #3:

How much Protein is in the control diet? Currently, only obese and lean are described. **This was meant to be included in Supplementary Table 1, which we made the mistake of omitting in the first submission. This is now included. The control diet contained 16.7% calories from protein.**

Figure 2: There is an interesting bi- or tri-phasic behavior in the foot pad swelling. Is there an explanation for this? It seems consistent in MAYV and CHIKV both. Also, line 255 says CHIKV and RRV, but only CHIKV is discussed. Was the data and discussion for RRV missed? It would be important to mention since E and F have a different mouse with RRV. Readers may be interested in the RRV C57bL/6 data for comparison. **The bi-phasic peak is commonly observed following alphavirus infection in immunocompetent mice. See Hease et al. for a review that discusses this briefly** [1]**. This is related to the immune response to the virus** [2,3]**. We did not infect C57BL/6 mice with RRV. We have attempted to clear up any confusion regarding which viruses were used to infect which mice. Thank you for catching this.**

Figure 4: B,C and D, labels difficult to see. What A represents is unclear. What ‘flux’ are we measuring? **We are sorry about the confusion. We have increased the size and moved the labels for B, C, and D. We have updated the figure legend of Figure 4, explaining that flux is measured in photons/second and that it is a measure of the amount of light emitted from the footpad.**

References.

1. Haese NN, Broeckel RM, Hawman DW, Heise MT, Morrison TE, Streblow DN. Animal Models of Chikungunya Virus Infection and Disease. J Infect Dis. 2016;214: S482–S487.

2. Dagley A, Ennis J, Turner JD, Rood KA, Van Wettere AJ, Gowen BB, et al. Protection against Chikungunya virus induced arthralgia following prophylactic treatment with adenovirus vectored interferon (mDEF201). Antiviral Res. 2014;108: 1–9.

3. Long KM, Whitmore AC, Ferris MT, Sempowski GD, McGee C, Trollinger B, et al. Dendritic cell immunoreceptor regulates Chikungunya virus pathogenesis in mice. J Virol. 2013;87: 5697–5706.
